# Copy number determination of the gene for the human pancreatic polypeptide receptor *NPY4R* using read depth analysis and droplet digital PCR

**DOI:** 10.1186/s12896-019-0523-9

**Published:** 2019-06-04

**Authors:** Kateryna Shebanits, Torsten Günther, Anna C. V. Johansson, Khurram Maqbool, Lars Feuk, Mattias Jakobsson, Dan Larhammar

**Affiliations:** 1 Department of Neuroscience, SciLifeLab, Uppsala University, Uppsala, Sweden; 2 Human Evolution, Department of Organismal Biology, SciLifeLab, Uppsala University, Uppsala, Sweden; 3 Department of Cell and Molecular Biology, SciLifeLab, Uppsala University, Uppsala, Sweden; 4 Department of Immunology, Genetics and Pathology, SciLifeLab, Uppsala University, Uppsala, Sweden; 5 Centre for Anthropological Research and Department of Anthropology and Development Studies, University of Johannesburg, Johannesburg, South Africa

**Keywords:** NPY4R, Copy number variation, Read depth analysis, Droplet digital PCR

## Abstract

**Background:**

Copy number variation (CNV) plays an important role in human genetic diversity and has been associated with multiple complex disorders. Here we investigate a CNV on chromosome 10q11.22 that spans *NPY4R*, the gene for the appetite-regulating pancreatic polypeptide receptor Y4. This genomic region has been challenging to map due to multiple repeated elements and its precise organization has not yet been resolved. Previous studies using microarrays were interpreted to show that the most common copy number was 2 per genome.

**Results:**

We have investigated 18 individuals from the 1000 Genomes project using the well-established method of read depth analysis and the new droplet digital PCR (ddPCR) method. We find that the most common copy number for *NPY4R* is 4. The estimated number of copies ranged from three to seven based on read depth analyses with Control-FREEC and CNVnator, and from four to seven based on ddPCR. We suggest that the difference between our results and those published previously can be explained by methodological differences such as reference gene choice, data normalization and method reliability. Three high-quality archaic human genomes (two Neanderthal and one Denisova) display four copies of the *NPY4R* gene indicating that a duplication occurred prior to the human-Neanderthal/Denisova split.

**Conclusions:**

We conclude that ddPCR is a sensitive and reliable method for CNV determination, that it can be used for read depth calibration in CNV studies based on already available whole-genome sequencing data, and that further investigation of *NPY4R* copy number variation and its consequences are necessary due to the role of Y4 receptor in food intake regulation.

**Electronic supplementary material:**

The online version of this article (10.1186/s12896-019-0523-9) contains supplementary material, which is available to authorized users.

## Background

Copy number variation (CNV) contributes greatly to the genetic variability in human populations. CNVs are a type of structural variation in the genome that vary in number and range in length from one kilobase to several megabases [1]. CNVs have been associated with many complex traits, including neurodevelopmental disorders [2, 3] and obesity [4–6].

**Fig. 1:**
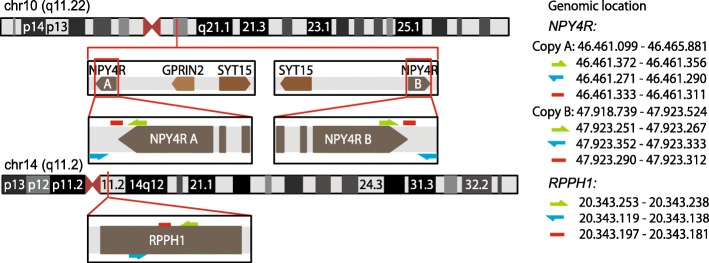
Chromosomal positions of *NPY4R* and *RPPH1* genes, and droplet digital PCR assay design. Green and blue arrows indicate primer positions, red lines indicate probe positions. The duplication region on chr10q11.22, as it is annotated in GRCh38 human genome assembly

Several research groups has described a CNV region on chromosome 10q11.22 [2, 5, 7–11] while, one study reports no CNV [12]. The region was initially reported to span almost 194 kb [13] across *NPY4R*, *SYT15* and *GPRIN2* genes (Fig. 1). This region is notorious for its complexity due to repeated elements and it has not been fully mapped in the current version of the human genome assembly (GRCh38). Most of the previous reports assumed that the normal copy number of *NPY4R* is 2 copies per genome [5, 9, 11–13] and describe the CNV as either gain or loss, not always specifying the exact copy number. Several studies have demonstrated that a CNV in this region is associated with differences in body weight [2, 5, 11, 13, 14].

*NPY4R* encodes Y4, a receptor for pancreatic polypeptide (PP). Due to the fact that PP is involved in appetite regulation, [15, 16] structural or functional changes in *NPY4R* might be of great importance for energy homeostasis and body weight.

Here we investigate CNV of *NPY4R* gene in 69 modern human samples from the 1000 Genomes project and of three archaic hominins (two Neanderthals and one Denisovan), using well-established computational methods of CNV detection by read depth analysis [17–20]. For 19 of the 1000 Genomes samples, we also determined the copy number of *NPY4R* using the recently developed molecular droplet digital PCR (ddPCR) method [21]. Our results differ from previously published studies of the CNV of this complex genomic region in that we find more copies and more variation with both of these approaches than previously published studies. The differences between the copy number determination methods are discussed.

## Methods

The aim of the study was to determine the copy number of the human *NPY4R* gene in samples from the 1000 Genomes database and to compare the computational read depth analysis methods with the ddPCR method in DNA from a subset of these samples. Finally, we performed read depth analysis of three archaic hominins.

### Samples

We have studied 66 samples from the 1000 Genomes Project [22] (see Additional file 1) and three archaic hominins (two Neanderthals and one Denisovan).

The low coverage samples from phase 1 of the 1000 Genomes Project were downloaded from the public repository, (http://www.1000genomes.org, download data 2012-06-01), then the copy number of the CNV region spanning *NPY4R* was assessed using CNVrd2 read depth analysis method. The phase 3 sequencing data for the same samples from the 1000 Genomes Project (*N* = 66) was analysed using three different read depth analysis methods, namely CNVrd2 [17] (except for NA18940), Control-FREEC [23] and CNVnator [24]. We have also investigated the high-coverage genomes of two Neanderthals and one Denisovan for their copy number in the *NPY4R* region using the Control-FREEC method. Finally, ddPCR was performed on 18 samples from the 1000 Genomes Project.

### Read depth analysis

#### CNVrd2

Chromosome 10 was extracted from the alignment for each sample and used for further analysis. The R-package CNVrd2 [17] was used to perform the actual read depth analysis and determine the copy number state of the CNV region spanning *NPY4R* for each sample. The results are summarized in Table 1.

**Table 1:** *NPY4R* CNV determined by read depth analyses

NPY4R copy number	Archaic hominins (*N* = 3)	1000 Genomes samples	
	Control-FREEC	Control-FREEC Phase 3 (*N* = 66)	CNVnator Phase 3 (*N* = 66)
1			
2			
3		1	1
4	3	50	50
5		12	12
6		2	2
7		1	1

Table showing comparison of the *NPY4R* copy number in 66 samples from the 1000 Genomes Project. Copy number was determined by three kinds of read depth analysis. Copy number is binned to the closest integer

#### Control-FREEC

In order to avoid spurious signals due to either the fragmentation in ancient DNA, differences in coverages or sequencing technologies, ancient samples were analyzed independently of each other and of the 1000 genomes data. BAM files mapped to the human reference GRCh37 were downloaded from the MPI-EVA, Leipzig (Denisovan [25]: http://cdna.eva.mpg.de/neandertal/altai/Denisovan/; Altai Neandertal [26]: http://cdna.eva.mpg.de/neandertal/altai/AltaiNeandertal/bam/; Vindija Neandertal [27], http://cdna.eva.mpg.de/neandertal/Vindija/bam/,). Control-FREEC was employed to call CNVs for the whole genome. First, samtools [28] was used to generate a mpileup file only including reads with mapping quality of at least 30. Then Control-FREEC was run with the following parameters: coefficientOfVariation = 0.05, breakPointThreshold = 0.6, breakPointType = 2. The results for the copy number state for the CNV region spanning *NPY4R* were extracted from the output. Non-integer values were obtained by multiplying the median ratio of local normalized read depth to global read depth with the ploidy (2).

#### CNVnator-based read depth

CNVnator [24] v0.3.3 was employed to investigate copy number state of the *NPY4R* region in 66 samples from phase 3 of the 1000 Genomes Project. We have scanned chromosome 10 (with a window size of 300 bp) for significant duplications overlapping a 200 kb CNV region that spans across the *NPY4R*. We have used chromosome 10 as a reference for copy number determination.

### Droplet digital PCR

Assays were designed according to the guidelines from Bio-Rad Laboratories. The primers and probe for *RPPH1* assay were: Forward: 5′-CGCGCGAGGTCAGACT-3′ Reverse: 5′- GGTACCTCACCTCAGCCATT-3′ Probe: 5′-(VIC)CCGGCGGATGCCTCCTT-3′. The primers and probe for *NPY4R* were: Forward: 5′- TGCATCCATTTGCATCG-3′ Reverse: 5′-CTGCAAGGCTTACTGTGCAC-3′ Probe: 5′- TCAGCTGTTTGTTCCTGGGAGAA(FAM)-3′. For primer and probe location see Fig. 1.

DNA was digested with BstXI restriction enzyme (10 U/μl, ThermoScientific, Cat#: ER1021) in buffer 0 for 1 h at 55 °C, followed by 20 min at 80 °C. A 22 μl mixture of 2 × ddPCR mastermix (Bio-Rad, Cat#: 186–3010), forward and reverse primers for target and reference assay (final concentrations of 900 nM each), probes for both assays (final concentrations of 250 nM each) and 15 ng of digested DNA was emulsified with Bio-Rad Droplet Generator Oil (Bio-Rad, Cat#: 186–3005) in a Bio-Rad QX100™ Droplet Generator (Bio-Rad, Cat#: 186–3001) according to the manufacturer’s instructions. The droplets were manually transferred to a 96-well plate (Eppendorf, Cat#: 951020362) and heat-sealed with Easy Pierce sealing foil sheets (Thermo Fisher Scientific, Cat#: AB-0757). Polymerase chain reaction was performed in a Bio-Rad C1000 thermal cycler (Bio-Rad, Cat#: 185–1197) with the following cycling parameters: 10 min at 95 °C (1 cycle), 30 s denaturation at 94 °C and 1 min annealing and extension at 58 °C (40 cycles), 10 min at 98 °C and a hold at 12 °C. All steps had a ramp rate of 2 °C/s. Droplets were analysed using a Bio-Rad QX100 Droplet Reader (Bio-Rad, Cat#: 186–3001). Fluorescent data from each well were analysed with QuantaSoft software (v1.3.2), where copy number was calculated based on Poisson distribution [29]. All DNA samples were run at least twice.

### Data analysis

The degree of correlations between *NPY4R* copy number data generated by read depth methods and average values of ddPCR were calculated using Spearman correlation (for *NPY4R* copy number data see Additional file 1). Statistical analysis was performed using SPSS version 22.0.

## Results

Using read depth analysis we have confirmed that *NPY4R* is located in a copy number variable region by analyzing 66 modern human samples from 1000 Genomes Project (for an example of a read depth output see Additional file 2: Figure S1, A). For simplicity, we will refer to the copy number of this CNV region as *NPY4R* copy number.

**Fig. 2:**
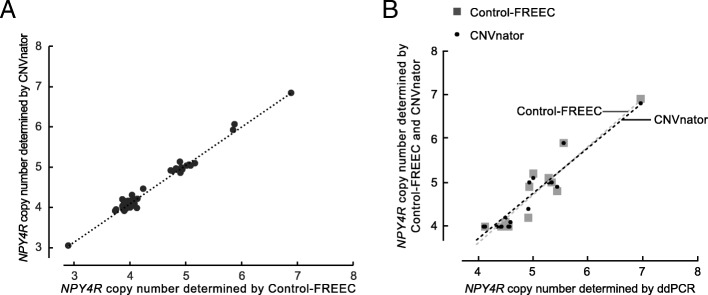
Results of read depth analyses of the *NPY4R* in 66 samples from the 1000 Genomes Project. Correlation between Control-FREEC and CNVnator results (all phase 3 data) (**a**). Results of ddPCR and read depth analyses of the *NPY4R* in 18 samples from the 1000 Genomes Project (**b**)

The copy number determined by the Control-FREEC and CNVnator methods ranged from three to seven among the 66 individuals with four as the most frequent copy number for the phase 3 data (Table 1). The results of the CNVrd2 method displayed no correlation with either the results of Control-FREEC or CNVnator analyses, nor with the ddPCR. The results of Control-FREEC and CNVnator displayed a statistically significant correlation but were not identical (Spearman’s ρ = 0.822, *p* < < 0.001) (Fig. 2a).

In addition to the read depth analysis described above, we have also analyzed the copy number of *NPY4R* by ddPCR for a subset of 18 samples from 1000 Genomes Project (Table 2) (for an example of a ddPCR output see Additional file 2: Figure S1, B).

**Table 2:** *NPY4R* CNV determined by read depth analysis and ddPCR

Sample ID	Population	*NPY4R* copy number		
		Read depth		ddPCR
		Control-FREEC Phase 3	CNVnator Phase 3	
NA10847	CEU	3.9	4.0	4.4
NA10851	CEU	5.9	5.9	5.6
NA12155	CEU	4.8	4.9	5.4
NA12717	CEU	3.9	4.0	4.1
NA18504	YRI	4.0	4.0	4.4
NA18510	YRI	5.1	5.0	5.3
NA18517	YRI	3.9	4.0	4.6
NA18519	YRI	5.2	5.1	5.0
NA18536	CHB	3.9	4.1	4.6
NA18542	CHB	4.1	4.2	4.0
NA18603	CHB	4.1	4.2	4.5
NA18627	CHB	4.0	4.0	4.1
NA18745	CHB	4.2	4.4	4.9
NA18940	JPT	6.9	6.8	7.0
NA18948	JPT	4.9	5.0	4.9
NA18949	JPT	4.0	4.0	4.6
NA18959	JPT	5.0	5.0	5.3
NA18961	JPT	3.9	4.0	4.3

Comparison of the *NPY4R* copy number in 18 individuals from the 1000 Genomes Project. Copy number was determined by two kinds of read depth analysis and ddPCR

Population acronyms: *CEU* Utah residents with Northern and Western European ancestry, *YRI* Yoruba in Ibadan, Nigeria, *JPT* Japanese in Tokyo, Japan, *CHB* Han Chinese in Beijing, China

Using the ddPCR method we observed a variation from four to seven copies of *NPY4R* per genome. The most frequent copy number was four. Both Control-FREEC and CNVnator displayed a correlation with the ddPCR results (Spearman’s ρ = 0.777, *p* = 1.488*10^−4^ and Spearman’s ρ = 0.818, *p* = 3.377*10^−5^ respectively) (Fig. 2b).

In order to investigate the archaic copy number state, we have analysed the NPY4R copy number in two Neanderthal and one Denisovan genomes. Both Neanderthals and the Denisovan had four copies of NPY4R (Table 1).

## Discussion

This study was designed to investigate the previously reported CNV for the *NPY4R* gene on chromosome 10q11.22 and study the range of the CNV. Earlier studies were contradictory with the majority of studies reporting CNV [2, 5, 7–11, 14] and one study describing no variation in this genomic region [12]. Here, we demonstrate that the *NPY4R* copy number varied depending on the analysis method and the quality of data. We had initially studied this CNV region using CNVrd2 on phase 1 data from the 1000 Genomes Project, which was the only data available at the time. Subsequently we have analysed the same samples from the 1000 Genomes Project with the CNVrd2 method, using the phase 3 sequencing data. The copy number determined by CNVrd2 in phase 1 and phase 3 data exhibit no correlation. The results of the CNVrd2 displayed no correlation with either the results of Control-FREEC, CNVnator or the ddPCR method, they were therefore considered to be untrustworthy (not shown). Using three independent methods (two types of read depth analysis and ddPCR) we demonstrate the presence of extensive CNV for *NPY4R*. Two read depth analyses (Control-FREEC and CNVnator) displayed a similar CNV range: three to seven copies, and ddPCR has shown a variation from four to seven copies (Table 1). We discuss below the methods for CNV determination and possible reasons for differences between reports of the CNV of this region.

### Differences between CNV determination methods

Read depth analysis was performed using genome re-sequencing data, where reads are mapped back to the reference assembly, and the resulting read coverage in each individual is used to determine the copy number of the region. A limitation of this approach is that any comparison between individuals is relative to the reference assembly, and estimates of copy number will be dependent on the number of copies present in the reference. For regions where the quality of the assembly is low due to e.g. a high frequency of repeats, errors in the reference assembly will affect the possibilities to accurately determine variation relative to the reference genome. When more specifically analyzing genomic short-read re-sequencing data for read depth differences, there is naturally a varying degree of read coverage over the genome that will affect the ability to accurately call copy number differences. While analyzing the results it is also important to keep in mind that the region of interest is small in itself and that we have used a small set of samples. Read depth methods are usually good at determining whether two samples differ in copy number relative to the reference genome. However, the absolute number of copies can be challenging to determine, especially if the copy number included in the reference genome is inaccurate or does not represent the population average. We initially used CNVrd2 to analyse the phase 1 data of the 1000 Genomes Project, where each individual was only sequenced to a moderate coverage, and different sequencing technologies (Illumina, 454 and SOLiD) were used (www.internationalgenome.org/analysis), lowering the accuracy of read depth copy number assignment. We have repeated the same type of read depth analysis on phase 3 data that was generated using the Illumina sequencing platform, had a higher and more even coverage. Due to lack of correlation between CNVrd2 analysis of phase 1 and phase 3 data, and absence of correlation between CNVrd2 and either Control-FREEC, CNVnator (all phase 3) or ddPCR, we concluded that CNVrd2 was unsuitable for analysing such complex regions, unlike, the two commonly used read depth methods that agree with each other and ddPCR.

There was no systematic difference, neither numerical nor ratio-wise, i.e., neither method systematically over- or under-estimates the number of copies relative to the other method.

The ddPCR method has recently emerged as an accurate way for precise quantification of target nucleic acid [21, 29]. It has been used for absolute copy number determination [30–32] and was shown to be equal [33] or more reliable than other molecular methods for copy number determination [34, 35] and comparable to other digital PCR methods [36]. The drawbacks of ddPCR are high cost and labour intensity in relation to number of studied CNV regions. In contrast, read depth analysis can determine multiple CNV regions [18] and estimate their copy number [17, 19], provided of course that the whole genome sequence is available (which is far more costly than ddPCR for a specific gene).

The shortcomings of both methods can be overcome by using ddPCR for calibration of read depth CNV estimation based on already available whole genome sequencing (WGS) data. Several studies have evaluated CNV regions based on read depth analysis of WGS data and ddPCR. Although the results were not identical, they demonstrated high concordance [20, 30, 37, 38]. Two of these studies have demonstrated that the difference between ddPCR and read depth analysis becomes grater with higher copy number [37, 38]. Taking into account the precision of ddPCR, we suggest that among the three read depth methods used in this study Control-FREEC and CNVnator are more precise copy number determination tools, and that ddPCR (as an absolute copy number measurement tool) can be used for evaluation and calibration of read depth-based CNV analysis of the WGS data.

Our ddPCR assays generated accurate and replicable results for both 1000 Genomes Project samples and in-house control DNA samples (see Additional file 3: Figure S2, A for showing the low variability of the reference assay between ddPCR runs and Additional file 3: Figure S2, B for comparison between two different reference assays). It is unknown whether, or to what extent, the propagation of immortalised lymphoblastoid cell lines has affected the integrity of their genomes and specifically the copy number state of *NPY4R*. For the purpose of methodological comparison, it would have been optimal to use DNA extracted from fresh blood samples in order to avoid possible effects of cell culturing. Nevertheless, due to high correlation between the read depth and ddPCR copy number estimates, we think that it is unlikely that transformation and propagation of the cell lines has influenced the *NPY4R* copy number.

### Accuracy of CNV determination is method-dependent

In comparison with previous studies that have reported CNV of the *NPY4R* gene, our analyses reveal both a generally higher copy number and greater variation. We find that the gene copy number ranges from three to seven (by Control-FREEC and CNVnator) (Table 1), and four to seven (by ddPCR) per genome (Table 2). Previously published studies assumed that the most common copy number of *NPY4R* is two copies per genome and did not involve accurate calibration to other genes [5, 9, 11–13]. That two copies was an incorrect estimate in most genomes was already obvious from SNP (single nucleotide polymorphism) frequency deviations from Hardy-Weinberg equilibrium observed in the 1000 Genomes Project samples (unpublished observations).

We have investigated the genomes of modern humans and archaic hominins in order to study the copy number state and time of the *NPY4R* gene duplication. As both of the investigated Neanderthal genomes and the Denisovan genome had four copies of *NPY4R*, which was also the most common copy number in modern humans (Table 1), we conclude that the NPY4R duplication took place before the split of modern humans from the Neanderthals and the Denisovans (400,000–800,00 years ago) [39].

One of the previous CNV studies could not detect any CNV in *NPY4R* region in young Chinese individuals [12]. We have analysed five individuals of Chinese origin using both of the read depth analysis methods as well as ddPCR, and we found *NPY4R* copy numbers either being equal to four (Control-FREEC) or ranging between four and five (CNVnator and ddPCR) (Table 2). Our results also agree with the most recent human genome assembly (GRCh38), where two copies of *NPY4R* are placed on the same chromosome, indicating that four copies per genome is the most likely normal copy number.

We suggest that the differences in *NPY4R* copy number described here and those reported in previous studies can primarily be explained by methodological differences (although, it is possible that population differences may also contribute). A common issue in CNV analyses is the need to make an assumption about the reference copy number. SNP-arrays, aCGH and RT-PCR-based methods require a reference copy number, which is most commonly set at two copies per genome [5, 9, 12, 13, 40]. We propose that incorrectly chosen reference copy number or inappropriate choice of reference gene in PCR-based copy number determination methods might be sources of error in CNV studies. We addressed this by performing three different kinds of read-depth analysis of chr10q11.22 region, which gave us a copy number relative to the rest of the chromosom 10 or the genome, as well as by using ddPCR, which gave us copy number relative to the reference gene. We observed that the most common copy number for *NPY4R* is four copies per genome. The haplotype in the current human genome assembly (GRCh38) shows two copies on the same chromosome, thus four copies per diploid genome. The analysis of CNVs in young Chinese individuals [12] was based on the assumption that the most common copy number for genes in the human genome is two and it used the *VEGFA* gene as a reference for copy number analysis by RT-PCR and presumed that this gene was a suitable reference. However, this gene has previously been shown to display CNV in an Asian cohort [9, 40, 41]. We have used *RPPH1* as a reference gene, since it is a commonly used well-known single-copy gene [42, 43].

Most of the previous studies were based on SNP arrays and aCGH, methods [2, 5, 7, 9, 13] that depend heavily on relative fluorescence data quality [44]. Signal intensity fluctuations of SNP arrays may occur as a result of probe length, the GC content of the probes and SNP position in the probe [45, 46]. DNA sequence-specific complications may cause poor probe coverage of certain genome regions, poor reproducibility and elevated risk of false detection. Algorithms for data analysis also differ in their sensitivity to the inherent variation in relative fluorescence between genomic loci on SNP and hybridization arrays [45, 46]. CNV genotyping assays often have a few target-specific probes located far apart, which makes these assays difficult to use with standard statistical methods that rely on the association between closely spaced probes [44]. Current genotyping platforms have limited or no probe coverage for a large number of common CNVs [47] and limited power to detect CNVs in duplication-rich and repeat-rich genome regions, such as chr10q11.22.

## Conclusions

In conclusion, our CNV study suggests that *NPY4R* varies in copy number and that the most common gene copy number is four per genome, not two as previously reported by other investigators. A comparative study would require many more individuals to draw conclusions at the population level and, especially, to investigate copy number differences between populations. Due to the CNV and the role of *NPY4R* and its ligand pancreatic polypeptide in the regulation of food intake, this gene is a strong candidate for contribution to body weight variation and obesity. However its exact role remains to be investigated, as the CNV in this region has shown both a positive and a negative correlation with BMI [5, 11, 13, 14]. We have demonstrated here that the quality of sequencing data plays a crucial role in read depth analysis and that methods for copy number determination can differ in precision. Based on multiple CNV studies [20, 30, 37, 38, 43, 48–50] as well as our own results we suggest that ddPCR is a reliable method for CNV determination that can be used to calibrate read depth analysis.

## Additional files

Additional file 1: *NPY4R* copy number in 1000 Genomes individuals. A table displaying IDs, population and the copy number of the *NPY4R* gene determined using read depth analysis (66 individuals from the 1000 Genomes database) and ddPCR (18 samples from the 1000 Genomes Project). (XLSX 14 kb)

Additional file 2: **Figure S1.** Methodology summary. Examples of read-depth data output with 6 and 4 copies of *NPY4R* (A). The notable low sequence depth of the regions surrounding the duplication unit in the Neanderthal genome is due to a lower sequence complexity of these regions (e.g. repeats), which makes them especially hard to map in the ancient genomes. An example of one ddPCR run (B). Red frames mark the samples displayed in B. Data presented with 95% confidence interval. (PDF 504 kb)

Additional file 3: **Figure S2.** Replicability of ddPCR measurements. *NPY4R* copy number measured in 3 samples (A). *NPY4R* copy number measurements based on two reference genes: *RPPH1* and *EIF2C1* (B). Data presented with 95% confidence interval. (PDF 128 kb)

## Abbreviations

aCGH: array comparative genomic hybridisation
BMI: Body mass index
CNV: Copy number variation
ddPCR: droplet digital PCR
PP: Pancreatic polypeptide
RT-PCR: Real-time PCR
SNP: Single nucleotide polymorphism
WGS: Whole genome sequencing

## References

[1] Feuk L, Carson AR, Scherer SW (2006). Structural variation in the human genome. Nat Rev Genet.

[2] Artuso R, Papa FT, Grillo E, Mucciolo M, Yasui DH, Dunaway KW (2011). Investigation of modifier genes within copy number variations in Rett syndrome. J Hum Genet.

[3] Gilman SR, Iossifov I, Levy D, Ronemus M, Wigler M, Vitkup D (2011). Rare de novo variants associated with autism implicate a large functional network of genes involved in formation and function of synapses. Neuron.

[4] Scherag A, Dina C, Hinney A, Vatin V, Scherag S, Vogel CIG (2010). Two new loci for body-weight regulation identified in a joint analysis of genome-wide association studies for early-onset extreme obesity in French and german study groups. PLoS Genet.

[5] Jarick I, Vogel CIG, Scherag S, Schäfer H, Hebebrand J, Hinney A (2011). Novel common copy number variation for early onset extreme obesity on chromosome 11q11 identified by a genome-wide analysis. Hum Mol Genet.

[6] Falchi M, El-Sayed Moustafa JS, Takousis P, Pesce F, Bonnefond A, Andersson-Assarsson JC (2014). Low copy number of the salivary amylase gene predisposes to obesity. Nat Genet.

[7] Wang K, Li W-D, Glessner JT, Grant SFA, Hakonarson H, Price RA (2010). Large copy-number variations are enriched in cases with moderate to extreme obesity. Diabetes.

[8] Sebat J, Lakshmi B, Troge J, Alexander J, Young J, Lundin P (2004). Large-scale copy number polymorphism in the human genome. Science.

[9] Park H, Kim J-I, Ju YS, Gokcumen O, Mills RE, Kim S (2010). Discovery of common Asian copy number variants using integrated high-resolution array CGH and massively parallel DNA sequencing. Nat Genet.

[10] Sudmant PH, Kitzman JO, Antonacci F, Alkan C, Malig M, Tsalenko A (2010). Diversity of human copy number variation and multicopy genes. Science.

[11] Aerts E, Beckers S, Zegers D, Van Hoorenbeeck K, Massa G, Verrijken A (2016). CNV analysis and mutation screening indicate an important role for the NPY4R gene in human obesity. Obesity.

[12] Sun C, Cao M, Shi J, Li L, Miao L, Hong J (2013). Copy number variations of obesity relevant loci associated with body mass index in young Chinese. Gene.

[13] Sha B-Y, Yang T-L, Zhao L-J, Chen X-D, Guo Y, Chen Y (2009). Genome-wide association study suggested copy number variation may be associated with body mass index in the Chinese population. J Hum Genet.

[14] Shebanits K, Andersson-Assarsson JC, Larsson I, Carlsson LMS, Feuk L, Larhammar D (2018). Copy number of pancreatic polypeptide receptor gene NPY4R correlates with body mass index and waist circumference. PLoS One.

[15] Asakawa A, Inui A, Ueno N, Fujimiya M, Fujino MA, Kasuga M (1999). Mouse pancreatic polypeptide modulates food intake, while not influencing anxiety in mice☆. Peptides.

[16] Batterham RL, Le Roux CW, Cohen MA, Park AJ, Ellis SM, Patterson M (2003). Pancreatic polypeptide reduces appetite and food intake in humans. J Clin Endocrinol Metab.

[17] Nguyen HT, Merriman TR, Black MA (2014). The CNVrd2 package: measurement of copy number at complex loci using high-throughput sequencing data. Front Genet.

[18] Mills RE, Walter K, Stewart C, Handsaker RE, Chen K, Alkan C (2011). Mapping copy number variation by population-scale genome sequencing. Nature.

[19] Handsaker RE, Korn JM, Nemesh J, McCarroll SA (2011). Discovery and genotyping of genome structural polymorphism by sequencing on a population scale. Nat Genet.

[20] Handsaker RE, Van Doren V, Berman JR, Genovese G, Kashin S, Boettger LM (2015). Large multiallelic copy number variations in humans. Nat Genet.

[21] Hindson BJ, Ness KD, Masquelier DA, Belgrader P, Heredia NJ, Makarewicz AJ (2011). High-throughput droplet digital PCR system for absolute quantitation of DNA copy number. Anal Chem.

[22] Locke AE, Kahali B, Berndt SI, Justice AE, Pers TH, Day FR (2015). Genetic studies of body mass index yield new insights for obesity biology. Nature.

[23] Boeva V, Popova T, Bleakley K, Chiche P, Cappo J, Schleiermacher G (2012). Control-FREEC: a tool for assessing copy number and allelic content using next-generation sequencing data. Bioinformatics.

[24] Abyzov A, Urban AE, Snyder M, Gerstein M (2011). CNVnator: an approach to discover, genotype, and characterize typical and atypical CNVs from family and population genome sequencing. Genome Res.

[25] Meyer M, Kircher M, Gansauge M-T, Li H, Racimo F, Mallick S (2012). A high-coverage genome sequence from an archaic Denisovan individual. Science.

[26] Prüfer K, Racimo F, Patterson N, Jay F, Sankararaman S, Sawyer S (2013). The complete genome sequence of a Neanderthal from the Altai Mountains. Nature.

[27] Prüfer K, de Filippo C, Grote S, Mafessoni F, Korlević P, Hajdinjak M (2017). A high-coverage Neandertal genome from Vindija cave in Croatia. Science.

[28] Li H, Handsaker B, Wysoker A, Fennell T, Ruan J, Homer N (2009). The sequence alignment/map format and SAMtools. Bioinformatics.

[29] Pinheiro LB, Coleman VA, Hindson CM, Herrmann J, Hindson BJ, Bhat S (2012). Evaluation of a droplet digital polymerase chain reaction format for DNA copy number quantification. Anal Chem.

[30] Boettger LM, Handsaker RE, Zody MC, S a MC (2012). Structural haplotypes and recent evolution of the human 17q21.31 region. Nat Genet.

[31] Beck J, Hennecke S, Bornemann-Kolatzki K, Urnovitz HB, Neumann S, Ströbel P (2013). Genome aberrations in canine mammary carcinomas and their detection in cell-free plasma DNA. PLoS One.

[32] Tayoun ANA, Mason-Suares H, Frisella AL, Bowser M, Duffy E, Mahanta L (2016). Targeted droplet-digital PCR as a tool for novel deletion discovery at the DFNB1 locus. Hum Mutat.

[33] Svobodová I, Pazourková E, Hořínek A, Novotná M, Calda P, Korabečná M (2015). Performance of droplet digital PCR in non-invasive fetal RHD genotyping - comparison with a routine real-time PCR based approach. PLoS One.

[34] Bharuthram A, Paximadis M, Picton ACP, Tiemessen CT (2014). Comparison of a quantitative real-time PCR assay and droplet digital PCR for copy number analysis of the CCL4L genes. Infect Genet Evol.

[35] Sillence KA, Roberts LA, Hollands HJ, Thompson HP, Kiernan M, Madgett TE (2015). Fetal sex and RHD genotyping with digital PCR demonstrates greater sensitivity than real-time PCR. Clin Chem.

[36] Dong L, Meng Y, Sui Z, Wang J, Wu L, Fu B (2015). Comparison of four digital PCR platforms for accurate quantification of DNA copy number of a certified plasmid DNA reference material. Sci rep. Nat Publ Group.

[37] Eisfeldt J, Nilsson D, Andersson-Assarsson JC, Lindstrand A (2018). AMYCNE: confident copy number assessment using whole genome sequencing data. Chan KYK, editor. PLoS One.

[38] Usher CL, Handsaker RE, Esko T, Tuke MA, Weedon MN, Hastie AR (2015). Structural forms of the human amylase locus and their relationships to SNPs, haplotypes and obesity. Nat Genet.

[39] Langergraber KE, Prüfer K, Rowney C, Boesch C, Crockford C, Fawcett K (2012). Generation times in wild chimpanzees and gorillas suggest earlier divergence times in great ape and human evolution. Proc Natl Acad Sci U S A.

[40] Park JH, Lee S, Yu HG, Kim J-I, Seo J-S (2012). Copy number variation of age-related macular degeneration relevant genes in the Korean population. den Hollander AI, editor. PLoS One.

[41] Liu MM, Agrón E, Chew E, Meyerle C, Ferris FL, Chan C-C (2011). Copy number variations in candidate genes in neovascular age-related macular degeneration. Invest Ophthalmol Vis Sci.

[42] Baer M, Nilsen TW, Costigan C, Altman S (1990). Structure and transcription of a human gene for H1 RNA, the RNA component of human RNase P. Nucleic Acids Res.

[43] Shoda K, Ichikawa D, Fujita Y, Masuda K, Hiramoto H, Hamada J (2017). Monitoring the HER2 copy number status in circulating tumor DNA by droplet digital PCR in patients with gastric cancer. Gastric cancer.

[44] Chu JH, Rogers A, Ionita-Laza I, Darvishi K, Mills RE, Lee C (2013). Copy number variation genotyping using family information. BMC Bioinformatics.

[45] B a T-P, Holliday EG, Evans T-J, McEvoy M, Attia J, Grice DM (2013). Continuing difficulties in interpreting CNV data: lessons from a genome-wide CNV association study of Australian HNPCC/lynch syndrome patients. BMC Med Genomics.

[46] Pinto D, Darvishi K, Shi X, Rajan D, Rigler D, Fitzgerald T (2011). Comprehensive assessment of array-based platforms and calling algorithms for detection of copy number variants. Nat Biotechnol.

[47] Cooper GM, Zerr T, Kidd JM, Eichler EE (2008). Nickerson D a. systematic assessment of copy number variant detection via genome-wide SNP genotyping. Nat Genet.

[48] Viljakainen H, Andersson-Assarsson JC, Armenio M, Pekkinen M, Pettersson M, Valta H (2015). Low copy number of the AMY1 locus is associated with early-onset female obesity in Finland. PLoS One.

[49] Abyzov A, Mariani J, Palejev D, Zhang Y, Haney MS, Tomasini L (2012). Somatic copy number mosaicism in human skin revealed by induced pluripotent stem cells. Nature. Nat Publ Group.

[50] Bonnefond A, Yengo L, Dechaume A, Canouil M, Castelain M, Roger E (2017). Relationship between salivary/pancreatic amylase and body mass index: a systems biology approach. BMC Med.

