# Supplementary material for: Copy number determination of the gene for the human pancreatic polypeptide receptor NPY4R using read depth analysis and droplet digital PCR
Source: BMC Biotechnol. 2019 Jun 4;19:31. doi: 10.1186/s12896-019-0523-9 (PMC6549351; doi:10.1186/s12896-019-0523-9)
Supplement: Supplementary file 2 — Figure S1. Methodology summary. Examples of read-depth data output with 6 and 4 copies of NPY4R (A). The notable low sequence depth of the regions surrounding the duplication unit in the Neanderthal genome is due to a lower sequence complexity of these regions (e.g. repeats), which makes them especially hard to map in the ancient genomes. An example of one ddPCR run (B). Red frames mark the samples displayed in B. Data presented with 95% confidence interval. (PDF 504 kb) [file 12896_2019_523_MOESM2_ESM.pdf]

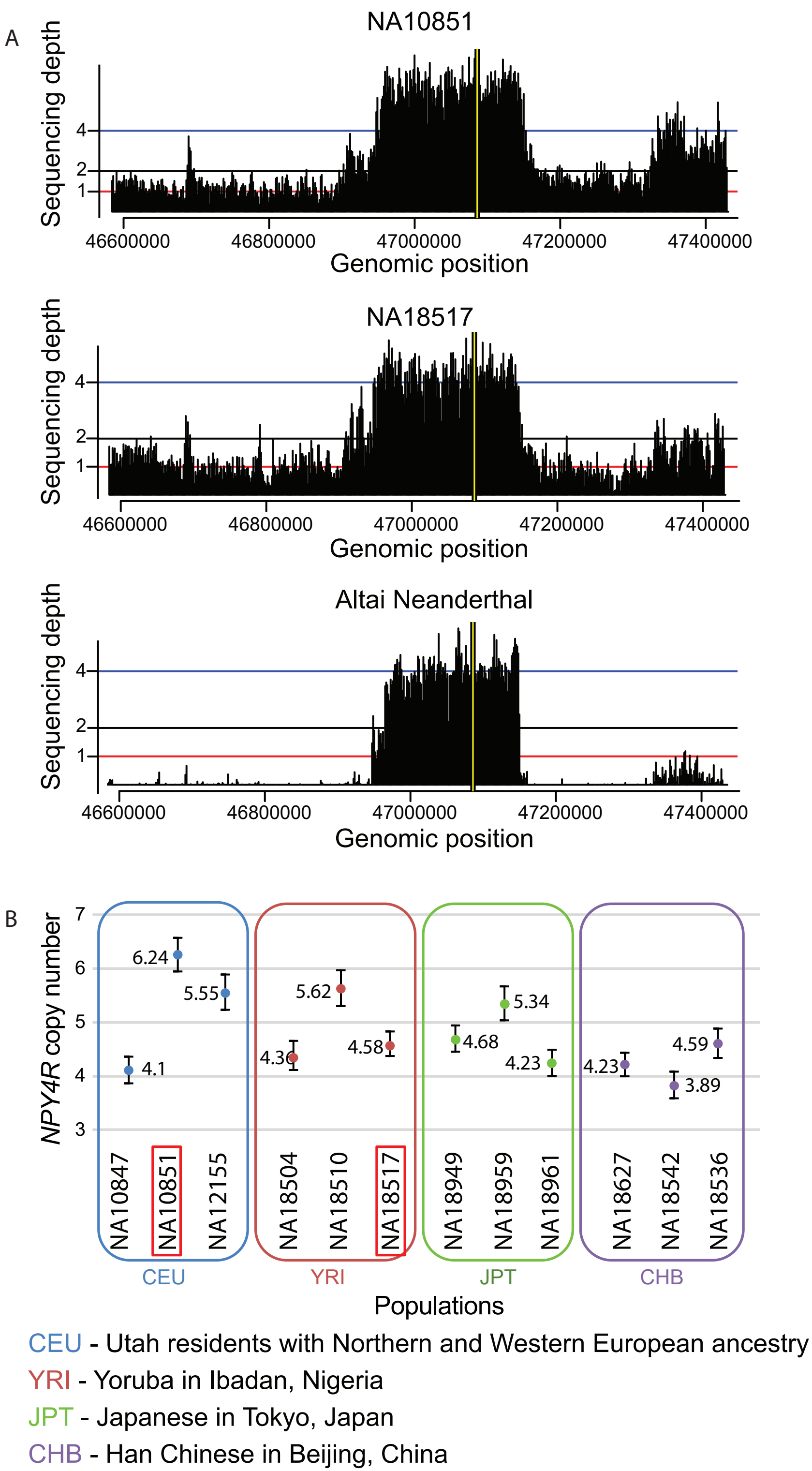
A
NA10851
Sequencing depth
4
2
1
46600000
46800000
47000000
47200000
47400000
Genomic position
NA18517
Sequencing depth
Genomic position
Altai Neanderthal
Sequencing depth
Genomic position
B
NPY4R copy number
7
6
5
4
3
6.24
5.55
4.1
5.62
4.36
4.58
5.34
4.68
4.23
4.23
4.59
3.89
NA10847
NA10851
NA12155
NA18504
NA18510
NA18517
NA18949
NA18959
NA18961
NA18627
NA18542
NA18536
CEU
YRI
JPT
CHB
Populations
CEU - Utah residents with Northern and Western European ancestry
YRI - Yoruba in Ibadan, Nigeria
JPT - Japanese in Tokyo, Japan
CHB - Han Chinese in Beijing, China
