# Supplementary figures and images for: Copy number determination of the gene for the human pancreatic polypeptide receptor NPY4R using read depth analysis and droplet digital PCR
Source: BMC Biotechnol. 2019 Jun 4;19:31. doi: 10.1186/s12896-019-0523-9 (PMC6549351; doi:10.1186/s12896-019-0523-9)

A

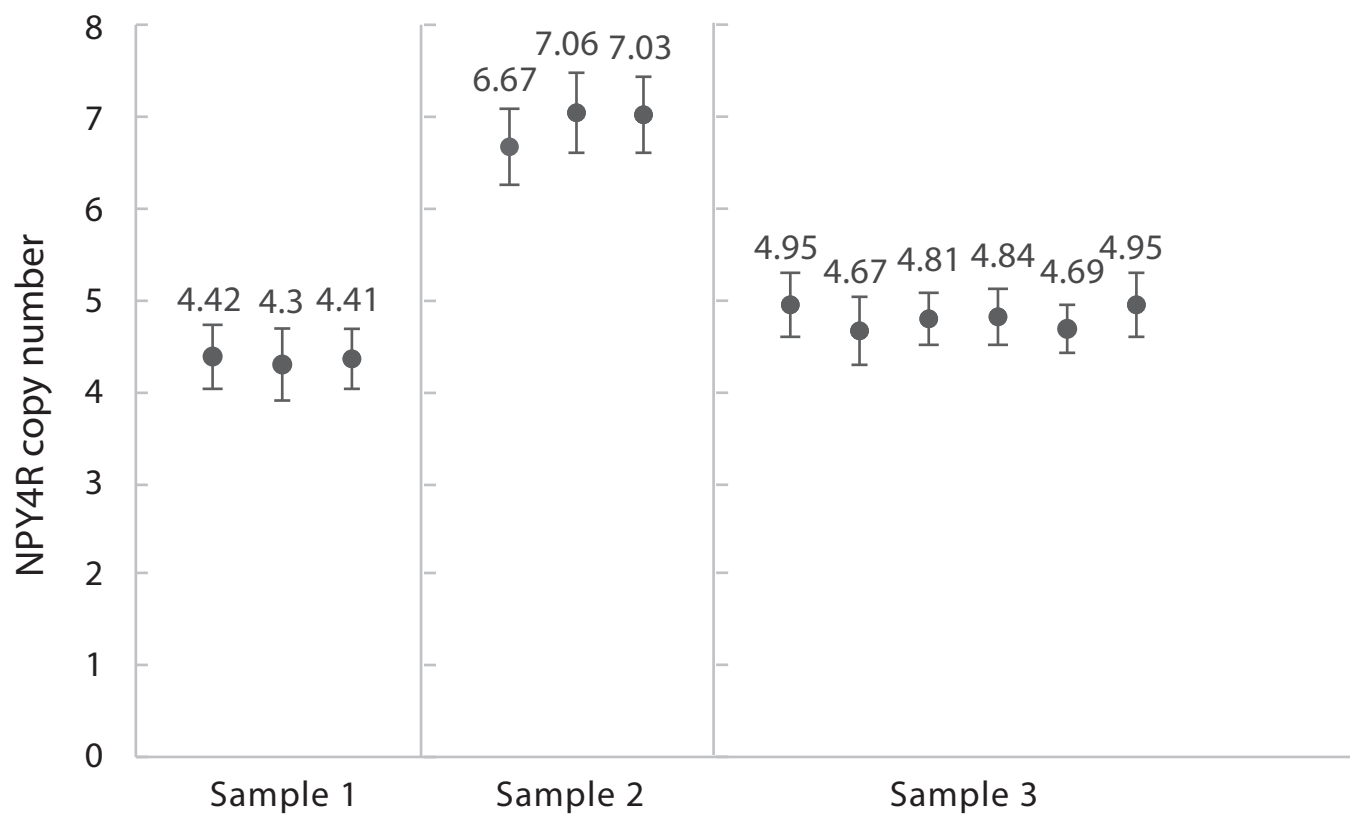

B

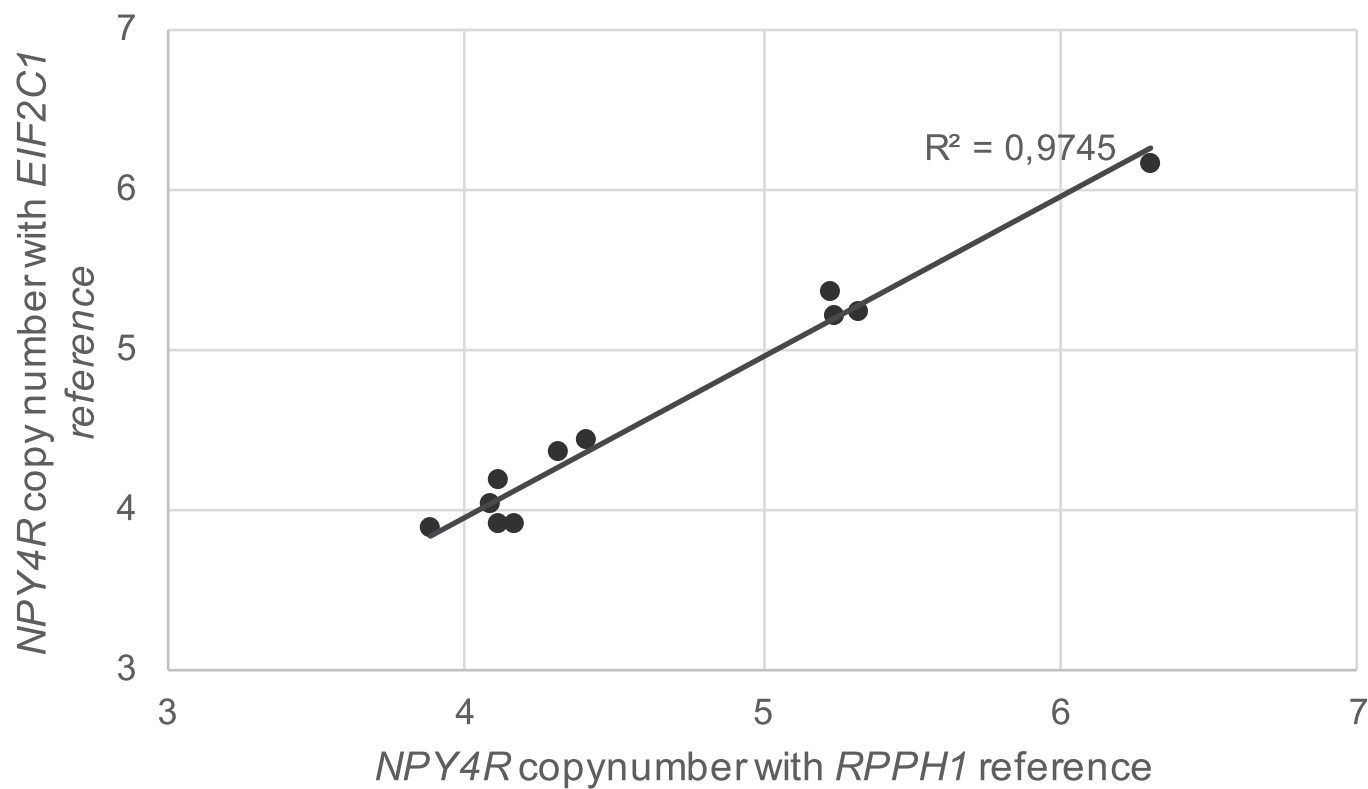

Supplement: Supplementary file 3 — Figure S2. Replicability of ddPCR measurements. NPY4R copy number measured in 3 samples (A). NPY4R copy number measurements based on two reference genes: RPPH1 and EIF2C1 (B). Data presented with 95% confidence interval. (PDF 128 kb) [file 12896_2019_523_MOESM3_ESM.pdf]
